# The visual politics of public police Instagram use in Canada

**DOI:** 10.1177/14614448211015805

**Published:** 2021-05-25

**Authors:** Kevin Walby, Blair Wilkinson

**Affiliations:** University of Winnipeg, Canada

**Keywords:** Community, public police, representations, rhetoric, silences, social media

## Abstract

Public police now use online and social media spaces as forums for communication. Drawing from discourse and semiotic analysis, and contributing to literature on police image management, we analyze police Instagram communications from five Canadian cities. Focusing on public police services’ Instagram posts, which are more indebted to visual communication than Twitter and Facebook, we examine the ways police communications frame community and diversity. Arguing that these communications resemble the fantastical authenticity found in other Instagram communications, we show how police mobilize images of community and diversity on Instagram to create positive affective relations with community. We argue that these communications amplify policing myths and operate to enhance police legitimacy. In the discussion, we assess what our findings mean for literatures on public police social media communications and policing myths.

## Introduction

Police communications are changing as technology advances and more questions are raised about police authority (Ellis, 2020; Lee and McGovern, 2013; Schneider, 2016; Wood, 2020). Public police now use online and social media spaces as forums for communication. Literature on this topic has examined the ethics and professionalism of police communications on social media (De Graaf and Meijer, 2019; Goldsmith, 2015), the diverse uses of social media by police (Hu et al., 2018), and the feedback loops that can exist between social media use and police practices (Schneider, 2016).

Each social media platform is unique and requires its own theorization and empirical investigation (Ellis, 2020; Murthy, 2012). The visual content of social media communication, exemplified by Instagram, requires examination as image making and sharing can have their own logic. Analyzing police Instagram communications from five Canadian cities, we extend analyses of the way that public police use images to maintain legitimacy (see Mawby, 2013). Focusing on public police services’ Instagram posts, which are more indebted to visual communication than Twitter or Facebook, we do so by examining the discursive tropes conveyed in these communications and also the semiotic positioning of police in relation to community and diversity. Hurley (2019) suggests social media is powerful because of the fantastical authenticity that it uses to communicate messages. Social media allows its users to “blur the lines between offline and online, fact and fiction, trust and deception, authenticity and fantasy” (Hurley, 2019: 13). Applying this idea in the criminal justice realm, the idea of fantastical authenticity lends itself to analysis of how police curate mythical images of themselves to boost their own legitimacy (Phillips, 2016). We draw from this work to examine the ideas and images that public police in Canada convey on Instagram.

First, we review literature on Instagram use and police communications. Second, we offer a note on research methods. Third, we present our analysis. We examine the myths conveyed through images and their associated narratives and strategic silences (i.e. what is not depicted, what is avoided) in police Instagram communications. We argue that the police communications specialists responsible for these communications strategically deploy myth and silence to construct a fantastically authentic version of their services and by extension boost their legitimacy by projecting to the public they are acting in socially acceptable and beneficial ways (Mawby, 2013; Wood, 2020). In the discussion, we assess what our findings mean for understanding public police social media communications and policing myths.

## Literature and theoretical framework

### Social media in context

Studying online communications is necessary to understand how information is exchanged in our digital world. While Facebook, Twitter, and YouTube are important to examine as major institutions turn to these media platforms to communicate with the public (Giglietto et al., 2012), Instagram deserves its own unique treatment and theorization (MacDowall and de Souza, 2018; Zappavigna, 2016; Zhao and Zappavigna, 2018) given its focus on the visual.

Choi and Lewallen (2018) show that Instagram users use pre-existing categories to make and sort their communications and creations, meaning their biases and prejudices can be incorporated into social media communications. As Gibbs et al. (2015) argue, “photo-sharing through Instagram echoes shifts in commemorative and memorialization practices, moving away from formal and institutionalized rituals to informal and personalized, vernacular practices” (p. 255). Vázquez-Herrero et al. (2019) argues that incorporation of social media such as Instagram into traditional media forums make media communication more ephemeral. Journalists end up chasing the image more than the story.

Social media is a forum for politics, and the complexity of image and text use online deserves academic scrutiny. Lalancette and Raynauld (2019) argue that Instagram can be used for image and message control by political entities. In the age of online friends and followers, digital and social media can allow affect to reverberate to audiences that would not otherwise be reached (Giglietto et al., 2012). Police social media is an affective strategy, and police are trying to make affective appeals that they could not while on patrol, serving warrants, or making arrests.

### Police communications and myth

Police culture is typically conceived of as authoritarian, white, and male. Chan (1996) critiques the notion of police culture for being uni-dimensional and insinuating the police are simply passive receptacles of a dominant police ideology. Police communications on social media must be understood as a way of amending traditional views of police culture in light of trends in community policing and calls for more inclusive organizations. Our research draws from literature on police communications and image management (McGovern and Lee, 2010; Mawby, 2013; Wood, 2020) to explore how public police in Canada attempt to revise traditional police culture, as well as engage in public relations work to manage their reputation.

A central dimension of police culture is the image of policing transmitted in news media (Donovan and Klahm, 2015) and social media. Police media units have long worked with newspaper and television media personnel to shape public views of policing (McGovern and Lee, 2010). Police openness to news media interviews has been indexed to accountability (Mawby, 2013). Public police try to manage the information that is known about their practices through media communication (Mawby, 2014) and our purpose is to examine these presentational strategies online. Social media is different since it is user-driven, with no journalists acting as gatekeepers of the messaging. Police social media use is planned by the police executive and media units (Ellis, 2020; Wood, 2020), and analysis of police social media communications provides some insights into the motivations of the police executive. As police have expressed anger about constantly being filmed and turned into popular cultural memes (Bayerl and Stoynov, 2016), police social media use attempts to take back images of policing and manage these their own way (Newell, 2019).

By exploring strategically selected images in police media presentations, this research contributes to literature on policing myths (Phillips, 2016). We understand policing myths as incomplete or idealized portrayals of policing practices which blur the lines between the authentic and fantasy and are, therefore, a form of fantastical authenticity (Hurley, 2019). For example, Russell (2018) examines the fusion of police imagery and LGBTI+ advocacy such as in construction of police as defenders of LGBTI+ persons and rights at Pride parades. These types of practices negate the history of repression queer people have experienced at the hands of public police (Russell, 2017). The myth conveyed is that police represent members of the public equally and conduct their work free of bias and stereotypes.

An equally important feature of social media communications and framing entails what is excluded from the image. Mason and Sayner (2019) consider the ways silence manifests in museum spaces, and we suggest this work on representations and silence is pertinent to analysis of police social media use as well. Silences can be strategic, operating as a form of camouflage or distraction (Dimitrov, 2017). Organizations can use silence as a way of preventing access to information about certain practices, past, and present. In the visual realm, with Instagram, these silences take the form of various occlusions that we examine below.

## Research methods

We are interested in trends in police social media use, and as qualitative researchers we are interested in the complexity of any single representation or image. What we provide here is an anatomy and visual archeology of police Instagram use (Airoldi, 2018). Data collection and analysis occurred in multiple stages. The first was a review of the Instagram pages of the five police services included in the study (Edmonton, AB; Ottawa, ON; Toronto, ON; Vancouver, BC; Victoria, BC). Often these images are curated by the personnel in police media relations offices. In this stage, images were reviewed to develop the main categories of analysis. The choice to only analyze images at this stage was purposeful as Instagram is an image-centered medium. We began the research with a goal to examine how the police use representations of masculinity or toughness to recruit. However, our initial analysis drew us to the eclectic nature of posts on these Instagram pages, which reflects the expanding scope of police responsibilities and the need for police services to be more inclusive. Thus, we began to understand that police were deploying alternative representations of their culture through Instagram and this yielded further insights into the data, resulting in the expansion of our coding categories used to select data. Our final coding categories were athleticism or prowess, community (i.e. community events and other community connections), diversity (i.e. BIPOC, 2SLGBTQQIA+, and other representations of diversity), dogs and other animals (a “genre” of Instagram posts demonstrated by the popularity of the #DogsOfInstagram), end of watch (commemorating fallen officers), police history, mental health, military (including associations with Canadian Armed Forces and invocations of para-military policing), patriotism, sports, protection (representing protection of public safety), recruitment and recruits, service (of community), teamwork, threat or danger, women in policing, and youth.

Data collection included retrieving screenshots of each post and collecting the narratives as text. We excluded wanted ads for two reasons. First, similar types of ads have been analyzed elsewhere (Lippert and Wilkinson, 2010). Second, unlike weapon or drug seizures which can be viewed as demonstrating protection of public safety, wanted ads demonstrate police failure to protect their communities. We also excluded public service announcements and safety advertisements unless these met another criterion for inclusion. A total of 200 posts (40 from each service) were selected for our dataset, with the most recent 40 image posts as of 26 October 2019 (the date data collection started) and those meeting our criterion being selected. Given key insights produced by similar posts, we also included three additional posts from Toronto Police and one video post from Vancouver Police related to 2SLGBTQQIA+ Pride to supplement our data on representations of diversity. The images and narratives were coded using an Excel spreadsheet. The data analysis was an iterative process. We continually refined our analysis, including through the writing process (Augustine, 2014), to arrive at understandings of what is communicated through police Instagram posts as well as the silences and absences in these communications. For example, our empirical focus on representations of community and diversity emerged only after we repeatedly encountered these themes and also noticed missing voices and accounts from these communities themselves.^1^

Data corresponding to our key themes were further analyzed using discourse and semiotic analysis. Using discourse analysis to examine captions and other wording allows us to reveal the common-sense ideas about social control animating these claims and police communications. Using this approach to assess the denotations and connotations of words, we examine the textual elements of these social media posts to assess what relationship, alliances, and allegiances are imagined (Janks, 1997). By focusing on the content, format, and context of these images, social semiotic analysis allows us to explore the visual imagery of the communications and interpret the representations used (Valverde, 2006: 28; Wilson and Landon-Hays, 2016). Social semiotic analysis helps us explore the visual rhetoric and politics in these communications, including how certain subjects are positioned and how certain icons and signs convey latent meaning. By using this approach to examining visual signification, we assess the symbolic meanings conveyed in these images including false or skewed connotations (Rose, 2016). Many of the images are curated to visually show positive affective relations (Lupton, 2020) between police and communities. Finally, we also conducted analysis of hashtags to explore key ways that police attempt to link their services to the broader community and engage in other communication strategies on Instagram.

## #OurTown: police communicating community

Although Instagram is a visual medium, it requires text-based methods for users to categorize their posts. Users categorize their posts by including hashtags (#), which can be used by other “Instagrammers” to search and follow posts they may find relevant or based on their location. Geo-location hashtags often use a city name or airport code. This hashtag search and follow function was leveraged by police to reach their audience and may leverage affective feelings of civic pride through connecting services with local communities. Edmonton, Vancouver, and Victoria frequently used this strategy, presumably to amplify the reach of their posts. #YEG (Edmonton’s airport code) was tied for the most prevalent hashtag with #VPD for Vancouver Police Department (*n* = 35 each); #YYJ (Victoria’s airport code) was the next most prevalent hashtag (*n* = 33). Other forms of city hashtags were also used. Toronto used #6ix (*n* = 10), signifying the six former municipalities that comprise Toronto after amalgamation, and Ottawa used #igottawa (*n* = 13) and #myottawa (*n* = 10).

Geo-based hashtags are not the only way for users to categorize their posts. Toronto Police Service created their own hashtag to promote their Enhanced Neighbourhood Community Officer Program (NCOP). Unlike geo-tagged posts, which locate the police services within their communities and extend the reach of their posts, Toronto’s #TOgetherWeAreToronto (*n* = 10) is self-promotional. This slogan relies on affective appeals as it creates a rhetorical tie between the Toronto Police Service and the Greater Toronto Area. This rhetorical linkage was not created solely through the hashtag, making it necessary for the @torontopolice account to develop an accompanying narrative that explained who was together and how they were together. Four of the posts using the hashtag brought the who (i.e. police and community) together by stating that Toronto Police Services’ “commitment to neighbourhoods is based on the understanding that we are not just IN the community, but truly connected to it” and stating that “[NCOP] is putting officers back on the ‘beat’ and entrenched in our neighbourhoods, benefiting our communities.” Yet, such claims conceal that this community connection is strained, as many Indigenous and Black Canadians are fearful of public police. This affective appeal is extended through six posts which create the composite image of an officer speaking with a member of the public and read, in part, “Our enhanced Neighbourhood Officer Program has 127 officers embedded in 35 neighbourhoods to *build partnerships and prevent crime* [emphasis added].”

Promoting policing initiatives is not the only way that police seek to demonstrate their connections to community through Instagram. Police attempt to accomplish this through “feel-good” posts about police making a difference in their communities. In a “repost” of a post from a Deputy Police Chief, Vancouver Police Department shows an image of a man in a fedora alongside the narrative:

Policing is a career that offers experiences beyond just arresting people & catching the bad guys. Policing gives us the opportunity to meet people in our community who are making a difference & changing the lives of those around them every day. Join #TeamVPD. We’re #Hiring. #VPD

Repost from Deputy Chow on Twitter:

Just learned about an officer who stopped a vehicle for an infraction a few months ago. Finds out driver teaches at #TempletonSecondary and was taking two of his underprivileged students to buy runners, because they needed them.

Cst. Mike Derry chose not to issue the ticket AND he gave $50 to the teacher to help buy some shoes for the kids. Way to go Mike and way to go Mr. Jimmy Crescenzo.

**Image 1.**
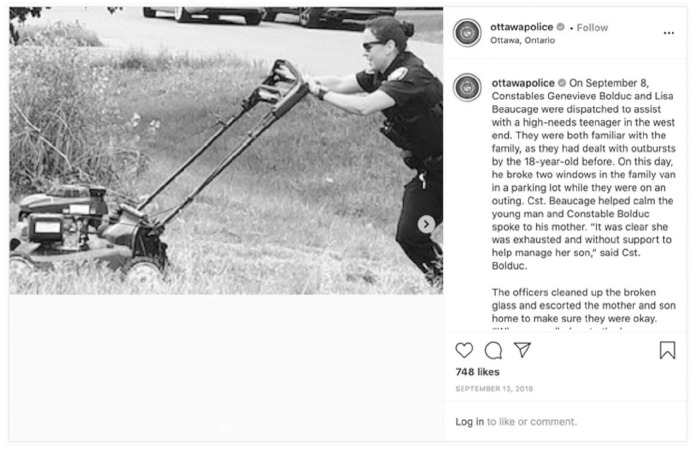
Ottawa Police officer mowing lawn.

In another post about officers serving community, Ottawa Police posted about officers assisting with cleaning up broken glass and mowing the lawn for the mother of a “high-needs teenager” following a call for the youth’s “outbursts” (Image 1).

These communications are performances of fantastical authenticity (Hurley, 2019) that tout myths about policing work. These feel-good stories use affective appeals to demonstrate police officers doing good in their communities. However, reliace on such images mute those police interactions that do not result in positive outcomes for people in the community. In the case of the Ottawa Police post, this silence depreciates those facing mental health challenges who have been killed in interactions with Ottawa Police officers (e.g. Greg Ritchie, Abdirahman Abdi). This silence also belies that 70% of the people who died during encounters with Canadian police had mental health issues, substance abuse issues, or both, and that 42% were in mental distress at the time of their death (Nicholson and Marcoux, 2018).

**Image 2.**
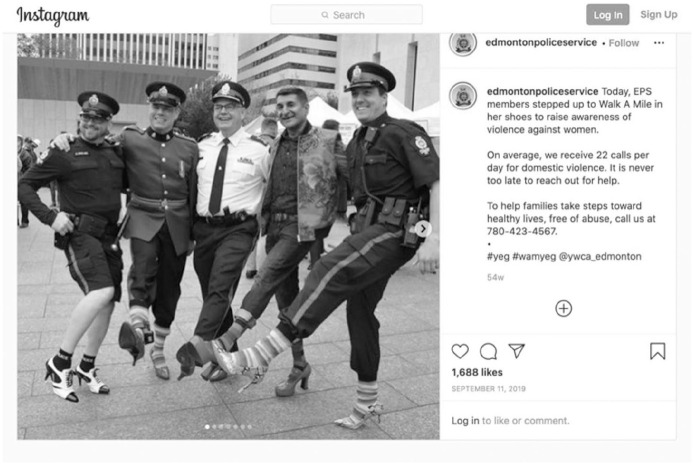
Edmonton Police service “Walk-a-Mile” post.

Additional “feel good” stories appear depicting police giving back to their communities through charity work. These posts included stories about charities widely associated with police services. The Special Olympics, in support of people with disabilities which is widely supported by police through the Law Enforcement Torch Run for Special Olympics, was promoted by Ottawa, Victoria, Vancouver, and Edmonton police services. Police-supported charity, Cops for Cancer (in support of the Canadian Cancer Society), was also promoted by Vancouver Police. Police Instagram accounts also promoted other charities that do not have as direct of links to police services. Edmonton Police Service promoted their officers’ participation in a Walk a Mile in Her Shoes event to “raise awareness of violence against women” (Image 2).

While recognizing the positive effects of “Walk a Mile” in raising awareness of violence against women, such posts present a fantastical authenticity where police relationships with gender non-conforming people who may experience fear and violence due to their gender expression, including for wearing heels like those worn by officers in the post, are not strained and Canadian police have not been criticized for their role in the crisis of missing and murdered Indigenous women, girls, and 2SLGBTQQIA+ people.

**Image 3.**
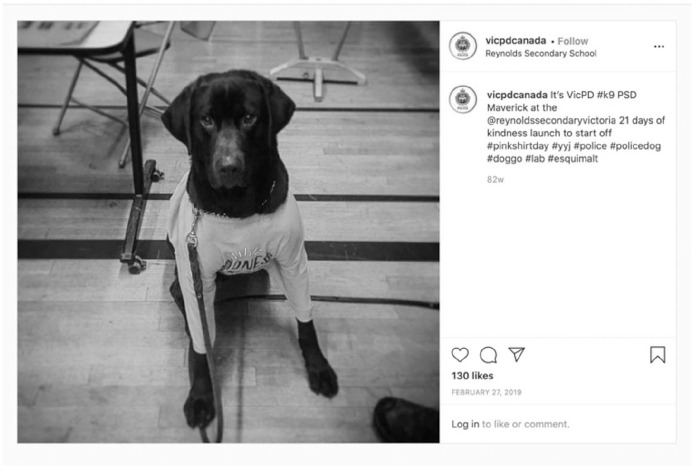
Victoria Police K9 promoting pink shirt day.

Police also promote their connections to community vis-à-vis youth. This includes through posts about charity events. For example, Victoria Police promoted their participation in events and support for Pink Shirt Day (Image 3). This day originated in Nova Scotia, Canada in support of a grade 9 boy who was bullied for wearing a pink shirt to school (CBC News, 2007). Victoria Police heavily promoted Pink Shirt Day, demonstrating their connections to and support for youth in their communities.

Despite being promoted by Victoria Police Department, these posts were devoid of messaging about how to stop bullying (i.e. not PSAs) and two posts did not even include reference to bullying. The lack of discussion of the focus of this program suggests that these posts are part of a public relations strategy that attempts to position police as protectors of youth (i.e. from bullying), while silencing experiences of those who are either fearful of police or believe they have been targeted or harassed by police due to their race (Regis, 2017).

**Image 4.**
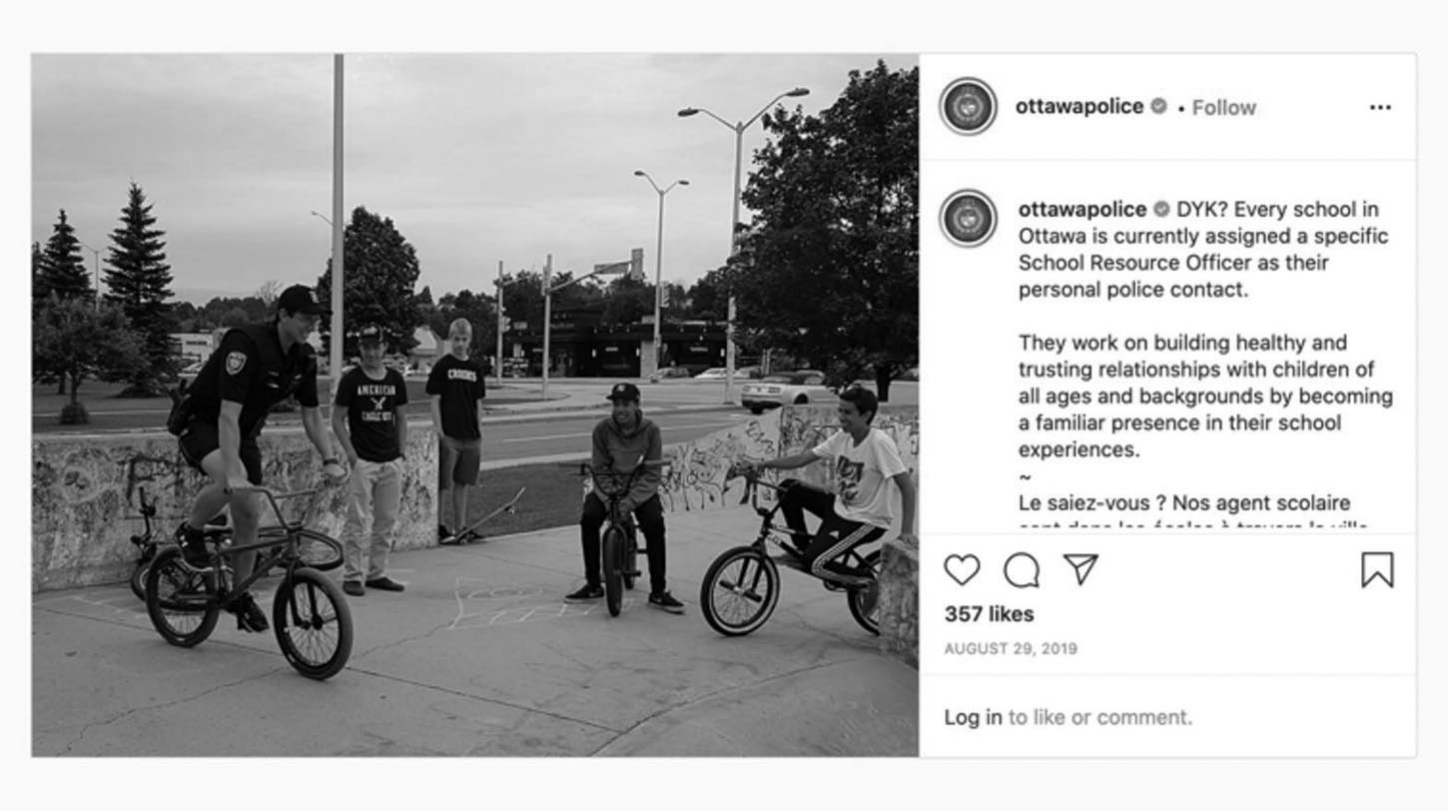
Ottawa Police school resource officer post.

A similar public relations-focus is elicited in posts by Ottawa Police Service that highlight their connections to youth and the value of these connections in fostering relationships. This includes a post promoting Ottawa’s School Resource Officer Program (Image 4) and another promoting an officer’s visit to an after-school program with the narrative reading in part with a message from the officer to youth that “The sky is the limit. Get involved in activities, join sports . . . Look for ways to make your community better. If you want to be a police officer, don’t be afraid to talk to them.”

Since youth-focused posts are meant to generate a positive affect and trust with police (Lupton, 2020), these types of posts can serve to legitimize police presence in schools. Such use of Instagram is intended to make the user appear more authentic and relatable (Reade, 2020). Hidden in such posts, however, are concerns about the need for and value of armed officers in schools, such as those described by Desmond Cole (2020) who outlines Black community resistance to armed police in Toronto area schools.

Posts about connections in the community attempt to legitimize police as part of their communities and are bolstered through appeals to police support of charities and protection of and service to youth. Posts promoting charities foster a mutually beneficial relationship as police seek legitimacy through their charitable giving while charities receive additional promotion for fundraising campaigns (Walby and Gumieny, 2020; Walby et al., 2018; Luscombe et al., 2018). Furthermore, appeals to connections to youth place police in a position of moral authority, indexing these communications to emotions associated with protection of youth. Police engage in public relations campaigns attempting to control the narrative about their services and mute criticisms, such as complaints by members of racialized and marginalized communities more likely to be subject to police violence.

## Representing diversity in policing

The importance of representation of underrepresented groups in media has been discussed in mainstream and academic circles. We analyzed our data to explore the following two main diversity-related categories: women in policing and diversity (claims of diversity, posts representing 2SLGBTQQIA+ people and events, Indigenous Peoples and events, and people of color). Diversity was evinced in 33/200 narratives and 64/200 images (or grouped images), while women in policing was represented in 23/200 narratives and 59/200 images (or grouped images). Representation of women, Indigenous people, and people of color in posts is important for police as these groups have been traditionally underrepresented in policing and due to police services’ fraught relationships with these groups (i.e. due to biased police practices as evinced through overrepresentation of Black and Indigenous people in criminal justice statistics and issues related to the policing of sexual assault accusations).

**Image 5.**
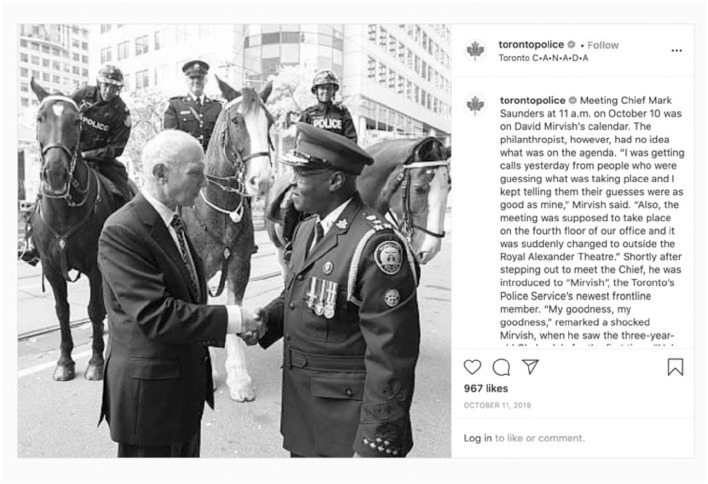
Toronto Police Chief with local philanthropist.

**Image 6.**
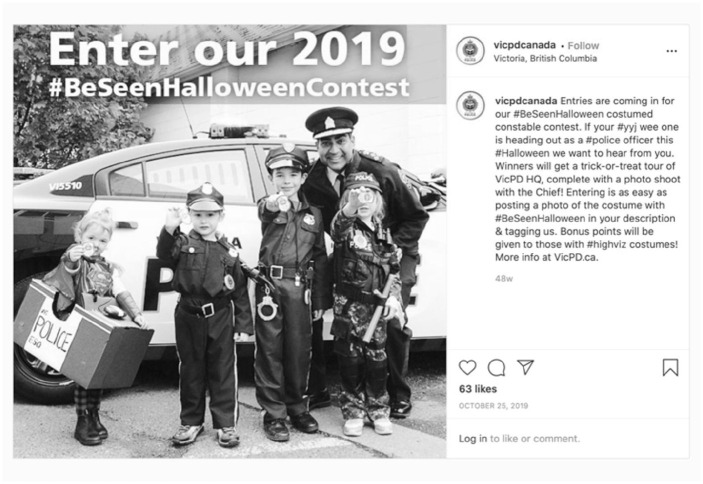
Victoria Police Chief halloween contest post.

For Victoria, Toronto, and Ottawa, representation of diversity in policing included those posts which had photos of their chiefs of police, two of whom are Black and one who is a person of color. Chiefs are spokespeople for their services and appear in numerous posts due to their participation in key events promoted on Instagram (Images 5,6).

These Chiefs become symbols of diversity in policing. While these representations are valid in that they demonstrate the ability for members of marginalized communities to rise through the ranks of police services, they obscure the challenges that members of these communities may experience in their professional roles. This includes having to witness or be subject to racist comments from other officers (Rigaux and Cunningham, 2020) or as was the case for Ottawa Chief Peter Sloly, being the target of a meme that uses a clip from the film “Downfall,” which compared Chief Sloly to Hitler and mocked his leadership and comments about racism in policing (Goodwin, 2020).

Attempts at representation of traditionally underrepresented groups are also demonstrated through recruitment-oriented posts. Recruitment-oriented posts are of two types: those which promote the recruitment of new officers and those that show new recruits. There is a juxtaposition in how these two types of posts represent diversity. Recruitment materials appear to feature greater representation of underrepresented groups (women and people of color) (Walby and Joshua, 2021), whereas those posts which show new recruits appear to show that women and people of color appear to remain underrepresented within actual practices (i.e. new recruits are largely white and male).^2^

These women were represented in less than 12.5% of narratives and only 30% of images is notable. The underrepresentation of women in policing is highlighted in a Toronto Police Service post which espouses the diversity of the service’s most recent graduating class of new officers. This post highlights the 20 women that are part of the class though the size of the class was 133 (TPS News, 2019). With respect to racial diversity, while this post claims that “[t]he graduating class is representative of Toronto’s diverse cultural community.” This appears to be misleading as the post identifies 36% of the class as being visible minorities, whereas 51.5% of people in Toronto are visible minorities according to the 2016 Census.

**Image 7.**
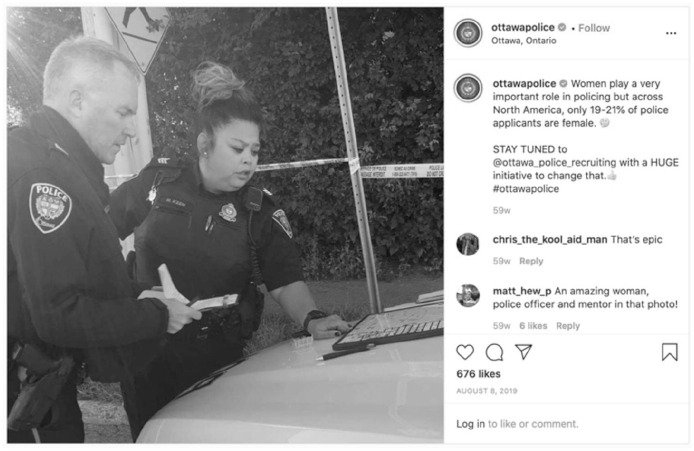
Ottawa Police recruiting announcement.

Police services attempted to recruit individuals from diverse backgrounds through their posts. For example, Ottawa Police Service acknowledged the underrepresentation of women in policing in a post, which states, in part, that “Women play a very important role in policing but across North America, only 19-21% of police applicants are female” and that Ottawa Police would undertake an initiative to change that (Image 7).

**Image 8.**
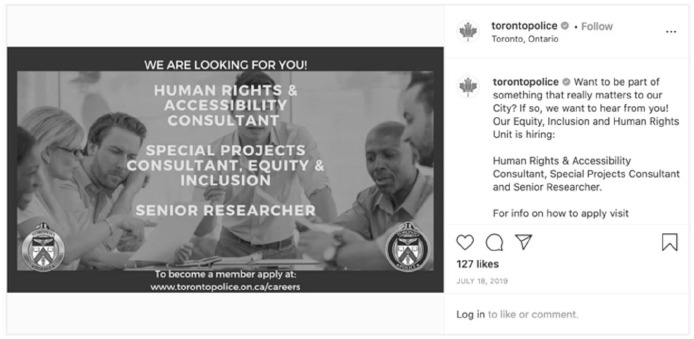
Toronto Police hiring advertisement.

Toronto Police Service also centered people from traditionally underrepresented groups in postings for a Data Collection Expert on a Race-Based Data Strategy Project (Image 8) and in their Equity, Inclusion and Human Rights Unit for Human Rights & Accessibility Consultant, Special Projects Consultant, and Senior Researcher. In a display of fantastical authenticity, both of these posts use stock imagery to represent diversity, rather than, for example, showing actual members of a diverse workforce. Following Zhao and Zappavigna (2018), these images and descriptions are not only a presentation of self or police subjectivity but are attempts to align the subjectivity of others with the police mission and extend authority and legitimacy of police.

**Image 9.**
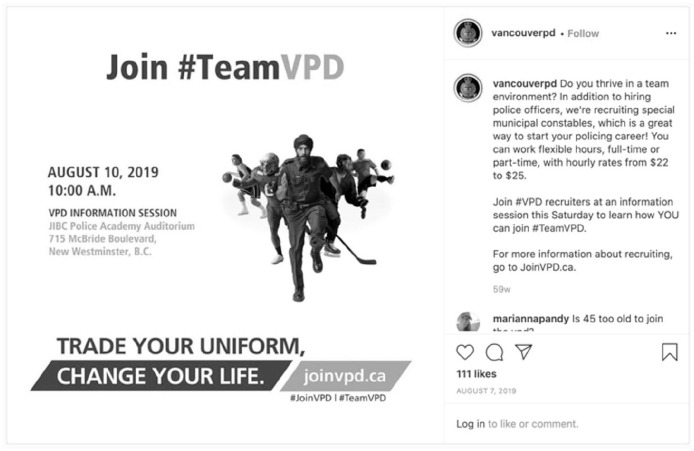
Example of #TeamVPD recruitment post.

Vancouver Police promoted the recruitment of people from traditionally underrepresented groups. This includes members of the 2SLGBTQQIA+ community through a repost from their department’s Recruiting Unit. Furthermore, someone who appears to be a Sikh man is centered in Vancouver’s “Join #TeamVPD” campaign that attempts to recruit former athletes to become members of the department (Image 9). While this campaign also appears to include two women, this campaign may further contribute to fewer women being recruited into the department as there is a gender gap in both sports participation in Canada (Statistics Canada, 2019) and opportunity in team sports (Donnelly et al., 2013: 3).

Diversity for police services may also be promoted not as diverse *cultural* backgrounds, but as diverse *experiential* backgrounds. As with the previous Toronto Police Service (TPS) post, which emphasized the backgrounds of new recruits including “previous military or policing experience and post-secondary education,” a post from Edmonton Police emphasized the “diverse backgrounds” of new recruits and focused on their previous jobs including “the armed forces, security, corrections, social services, health care, business, trades, music, and sports.” This focus on diversity of experiences may mythologize police diversity through reinforcing a definition of equity that does not contribute to eliminating barriers for underrepresented groups in policing.

## Representing #PrideInPolicing

The police relationship with the 2SLGBTQQIA+ community has been marked by oppressive police tactics, such as the policing of men who have sex with men through bathhouse surveillance and arrests (Walby, 2009). These tactics are evident in the historical relationship between police and the 2SLGBTQQIA+ community in Victoria, as demonstrated by threats of arrest for nudity leveled at women who participated bare-breasted in the 1996 Gay Pride Parade (Island Capsule, 1996). Through their Instagram communications, police services are attempting to demonstrate their connections to and support for their cities’ 2SLGBTQQIA+ communities in ways that obfuscate tensions between members of these groups.

**Image 10.**
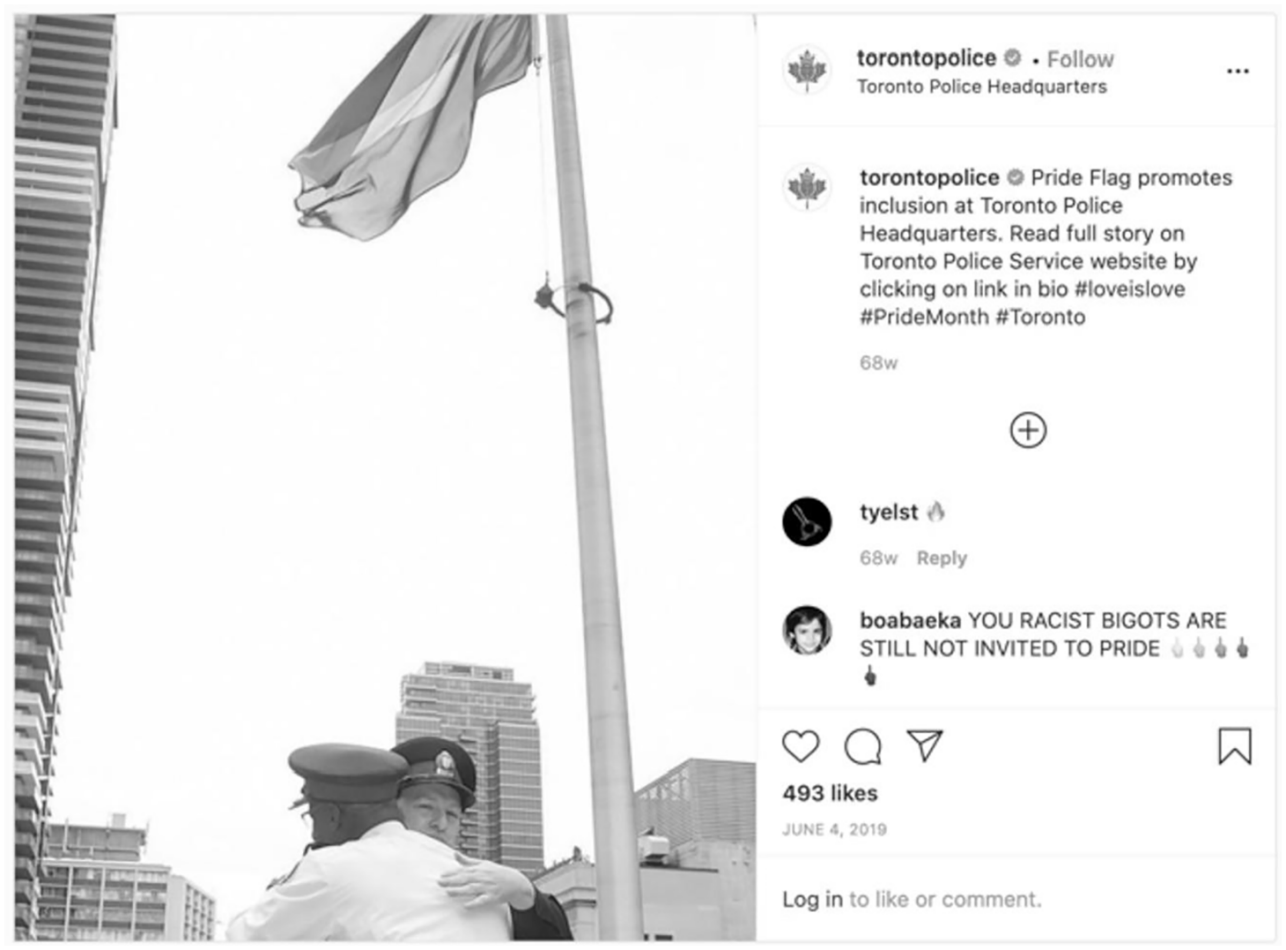
Example of Toronto Police Pride post.

Our social media data for Ottawa, Toronto, Vancouver, and Victoria include police participation in or support for Pride Week events put on by members of 2SLGBTQQIA+ communities in their jurisdictions. Toronto, Vancouver, and Ottawa promoted Pride events through posts about the raising of Pride flags. Vancouver’s post includes a slow-motion video which shows a flying Pride flag. The Pride flag is shown in the foreground of the video in front of the Canadian and Vancouver Police flags. Toronto Police Service shows the Pride flag displayed at Police Headquarters in a series of posts, including that shown in Image 10 in which two officers are seen embracing under the Pride flag, with the caption:

Pride flag promotes inclusion at Toronto Police Headquarters. Read full story on Toronto Police Service website by clicking on link in bio #loveislove #PrideMonth #Toronto

**Image 11.**
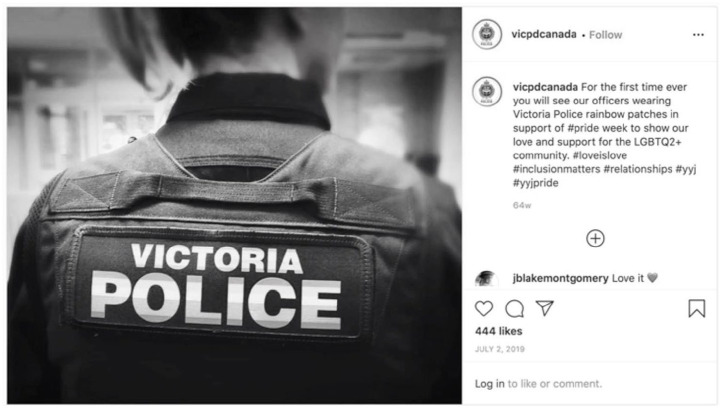
Victoria Police Pride post.

Ottawa Police also showed their connections with Pride through their post showing officers attending the flag raising at Ottawa’s City Hall. This post included a series of four photos, with the principal photo (and two others) showing officers in the foreground with the Pride flag in the background and the narrative: “Celebrate with Pride! I Célébrez avec fierté! #myottawa #ottawapolice #ottawaig #igottawa #pride #ottawapride.” Victoria Police did not show their officers attending a Pride flag raising, or a Pride flag raised at their police headquarters. Rather, the Victoria Police post focused on that service’s introduction of a rainbow patch worn by their officers for the first time (Image 11).

This patch appears to be a trend within policing to “rainbow-wash” or “rainbow-brand” (Russell, 2018) their image through adding rainbow versions of their services’ logos or other police-worn items. The wearing of the patch on the uniform is noteworthy in the context of requests by Pride organizations for police to no longer wear uniforms in the Pride Parade or to not be present at all at Pride Parades.

In several posts related to Pride, there is a focus on “love” through the use of the hashtags #lovewhoyouwant (Vancouver) and #loveislove (Victoria, Toronto). “Love is love” as a slogan used in the fight for marriage equality has been criticized as a form of respectability politics, which defines queer existence in relation to heteronormative ideals (Demir, 2019). Police’s use of these slogans is noteworthy in the context of policing’s historical and continuing relationships with the 2SLGBTQQIA+ community. Exclusive use of #loveislove and #lovewhoyouwant reduce the struggles of the 2SLGBTQQIA+ community to matters related to relationships and ignore the myriad other forms of oppression experienced by this community.

A brasher silence appears on Edmonton’s Instagram page. In May 2019, Edmonton’s Chief of Police issued an apology to members of the 2SLGBTQQIA+ community for “a history of abuse and mistreatment of the community by officers, referring to police raids at bath houses frequented by gay men, mistreatment during arrests and public shaming” (Short, 2019). Given the importance of this apology, one may conclude that the Police Service would wish to reach the largest audience, and particularly younger members of the 2SLGBTQQIA+ community who may be more likely to use Instagram than other forms of social media. However, a review of posts in May 2019 reveal no such use of Instagram for this purpose. This is surprising considering that Edmonton’s Instagram comprised a large number of #throwbackthursday posts which the service uses to craft a positive image of police, a use of Instagram that aims to entrench police legitimacy. That Edmonton Police would issue a public apology but not post this to their Instagram demonstrates how posts are strategically selected to portray positive representations of policing (see Redd and Russell, 2020).

Through wearing the patch on the uniform and promoting themselves on Instagram, the Police insert themselves into Pride as they are being asked by some members of the community to remove themselves from what emerged from the Stonewall Riots directed at unjust police practices. The image and narrative serves to silence legitimate complaints against police and their participation in Pride events. As Russell (2017) notes, the image “becomes a means of legitimizing past policing practices with the aim of overcoming poor and antagonistic LGBT-police relations” (p. 277). It is also contrary to what research shows about heterosexism and police culture. Gay and lesbian officers have experienced discrimination (Colvin, 2009; Jones and Williams, 2015). Many gay and lesbian officers must stay closeted or manage their identity carefully given the authoritative culture of police (Bernstein and Kostelac, 2002; Miller et al., 2003). While police posts may show authentic attempts for police to be more inclusive of 2SLGBTQQIA+ officers and community members, these mythologize relations as positive and silence negative experiences of 2SLGBTQQIA+ people.

## Promoting connections with Indigenous peoples

A lack of discussion of the relationship between police and Indigenous Peoples in Canada, with respect to historical injustices (e.g. Royal Canadian Mounted Police’s (RCMP) role in residential school apprehensions, police role in enforcing the pass system) and contemporary injustices (e.g. police handling of missing and murdered Indigenous women, girls and 2SLGBTQQIA+ people (MMIWG2S+); police surveillance of Indigenous activists), is another feature of these social media communications. Police posts on their connections to and support for Indigenous Peoples are largely ahistorical, with major silences regarding the role of police in reinforcing colonialism and the genocide of Indigenous Peoples (National Inquiry into Missing and Murdered Indigenous Women and Girls (Canada), 2019).

**Image 12.**
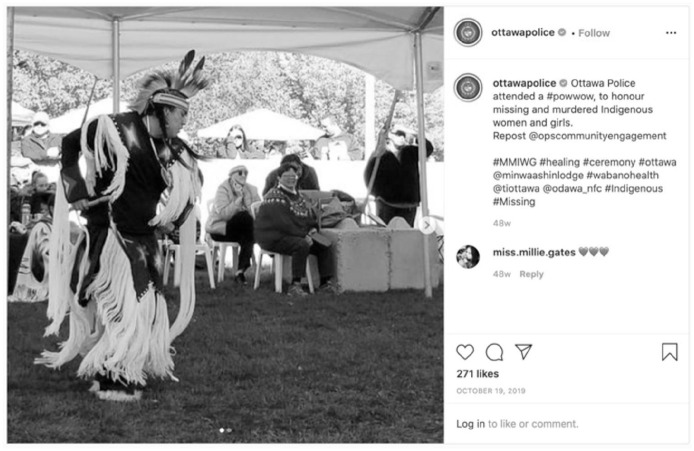
Ottawa Police MMIWG post.

Two posts stand out for their silences with respect to police-Indigenous relationships due to their glaring silence about police services ongoing role in colonization and its negative impacts while emphasizing reconciliation. In this post (Image 12), Ottawa Police promoting their attendance at a Pow Wow is most glaring as there is no recognition from the Police that this report targeted police (in)actions that have contributed to the number of missing and murdered Indigenous women, girls and 2SLGBTQQIA+ people in Canada.

**Image 13.**
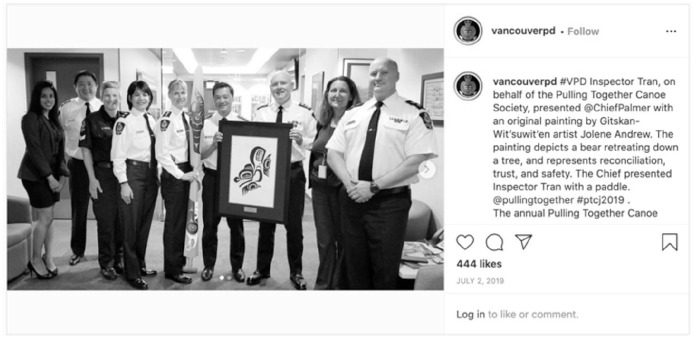
Vancouver Police pulling together canoe post.

Similarly, posts from the Vancouver and Victoria police services both speak to reconciliation. Vancouver Police speak to reconciliation in a post about their participation in the Pulling Together Canoe Journey and the presentation of a painting that “depicts a bear retreating down a tree, and represents reconciliation, trust, and safety” (Image 13).

In a post from Victoria Police, showing police and members of the “Aboriginal Street Community” at a Western Hockey League game, the narrative reads,

Thanks to the @victoriaroyals for hosting us and members of the Aboriginal Street Community. Our officers & staff really loved sitting down with members of the community we serve to take in some great @westernhockeyleague hockey! This is one small step on the journey towards #reconcilliation we are taking together. #yyj #goroyals #royalblue #community #police #reconcilliation #aboriginal #indigenous #hockey #healing.

Both posts remain silent on certain truths about colonization in Canada that is a necessary acknowledgment on the path to reconciliation. For example, the posts remain silent on police services’ role in colonization that have necessitated attempts at reconciliation through attempts at relationship building, and that British Columbia is almost entirely unceded Indigenous territory. The communications are also silent about the policing and arrest of Indigenous land defenders and water protectors throughout Canada.

Posts such as the ones above and others including a Vancouver Police Department post that promotes their “thankful[ness] to be part of such an inclusive #community” overlook continuing police practices that are part of the ongoing marginalization of Indigenous Peoples, such as Vancouver Police’s arrest of an Indigenous man and his 12-year-old granddaughter who were attempting to open up a bank account and for which the chief of police defended the handcuffing of the child (Ballard, 2020). These images are attempts to construe relationships between police and social groups that are absent in many real police-citizen encounters (also see Zappavigna, 2016). While the posts and the actions they depict may be authentic, showing attempts at reconciliation, by not acknowledging their role in ongoing and historical colonial practices or discrimination police mobilize Instagram to craft a myth of police as *only* having positive relationships with Indigenous people. This has the effect of presaging that their path to reconciliation is not being disrupted by ongoing colonial practices and police discrimination.

## Discussion and conclusion

Social media platforms such as Instagram allow users to curate their identities. Our data suggest that police services are using Instagram to curate narratives about their community focus and embracing of diversity. Like the Instagram user who curates their profile with pictures of their vacations and other socially desirable activities, while excluding images of them toiling at their day job, the police communications we examined are strategically curated.

Through their selection of images and crafting of narratives, these police services curate “myths” to reinforce organizationally acceptable stories. Our analysis reflects what Hurley (2019) refers to as the fantastical authenticity of social media communications, since the Instagram communications appear authentic given the use of images but are in equal part fantastical and mythical. As Aiello (2014) argues in an analysis of representations of police in popular culture, these communications convey a mediated ideal of police. This mediated ideal is out of touch with research and community views that understand police to be a violent force in society (Dobchuk-Land, 2017) that creates rather than mitigates harm. Thus, such images on social media convey myths regarding criminal justice personnel and organizations (Valverde, 2006). These attempts at producing ideal images of police and boosting police legitimacy cannot be disentangled from the violent histories of law enforcement and criminal justice (Wall and Linnemann, 2014). It may be the case that police are working to create better relationships with certain social groups (e.g. Indigenous Peoples and 2SLGBTQQIA+ peoples), yet the carefully managed and curated profiles silence parts of those relationships (or lack thereof) with certain members of these groups and distracts from the acts of violence and oppression that police continue to perpetrate against these groups in Canada and elsewhere.

We have examined myth making and communicative silences in police use of Instagram. It is not surprising that police curate their personas in this way, as these forms of communication facilitate a scope and resonance that police did not have with traditional media. In this way, police are able to bypass the news media, which may be more apt to present counter-narratives to these myths conveyed by the police. While social media can be used by those silenced to address these myths, these people and groups often lack the resources, such as staffed police media relations units, to amplify their voices. By using Instagram to create these myths, police may be able to influence public opinion. Where people are able to voice their opinion about police misconduct, including through social media resistance (see Hayes (2017) or Jackson and Foucault Welles (2015) on the #myNYPD social media protest), the police are constantly waging a social media campaign to persuade the general public into viewing police positively. With constant messages about police attempts to create positive relations with certain groups, the general public may be more inclined to accept that enough is being done and that those expressing dissatisfaction with police are “career activists” or over-exaggerating their claims.

Police are using social media to construct myths (Phillips, 2016), including through their silences (Mason and Sayner, 2019), about their commitment to progressive change within their organizations in an attempt to bolster their legitimacy. These visual displays operate to create a sense of positive affective relations between police and various communities (also see Brown and Carrabine, 2019). The need to construct their fantastical authenticity using social media suggests that police grasp on their legitimacy remains tenuous and this is made clear through continued resistance to the police (e.g. Defund the Police, Black Lives Matter). What remains uncertain is the success that online activists may have in overturn these policing myths using their own creative social media communications. Instagram can be used for resistance and organizing, although it remains to be seen if the communications and images of policing will be successfully contested in the social media age.

## Notes

1 Recognizing that one’s positionality informs data analysis, initial coding was conducted by Author 2, a white, queer settler and further data analysis was conducted in conjunction with Author 1, a white, cisgender settler.

2 Our analysis of representation of race and gender in images is limited. This analysis relies on the authors’ subjective identification of those represented (e.g. some individuals who we coded as “white” may self-identify otherwise). We suspect that police would promote their hiring of non-cisgender officers to show diversity (e.g. Toronto Police Service (TPS) hired first openly transgender officer in 2019 (Mauracher, 2019)) and the lack of promotion meant we coded gender as male or female, despite our understanding that some officers may identify their gender in another way.
